# Endoscopic vacuum therapy of a refractory esophagopleural fistula in a patient with Boerhaave syndrome, using an innovative hybrid stent

**DOI:** 10.1055/a-2363-8977

**Published:** 2024-08-07

**Authors:** Ivo Mendes, Gonçalo Nunes, Francisco Vara-Luiz, João Vaz, Carlos Luz, Jorge Fonseca, Pedro Pinto-Marques

**Affiliations:** 1 70816 Gastroenterology, Hospital Garcia de Orta EPE, Almada, Portugal; 2 Aging Lab, Egas Moniz Center for Interdisciplinary Research (CiiEM), Egas Moniz School of Health & Science, 2829-511 Almada, Portugal; 3 70816 Surgery, Hospital Garcia de Orta EPE, Almada, Portugal

Esophageal perforation has a high morbidity and mortality 1 . Endoscopic treatments include the use of self-expanding metal stents (SEMSs) and endoscopic vacuum therapy (EVT). Recently, an innovative hybrid stent combining both approaches, the VAC Stent (MicroTech), has been approved 2 .

A 45-year-old man was admitted with Boerhaave syndrome. Thoracic CT showed acute mediastinitis with bilateral pleural effusion. Upper endoscopy confirmed a 6-mm transmural defect in the distal esophagus that was treated using a 12 × 23-mm fully covered self-expanding metal stent (FCSEMS). Although the stent stayed in place the patient remained septic after 2 weeks. Oral contrast-enhanced computed tomography (CT) and methylene blue drainage into the right chest tube suggested persistent esophagopleural fistula. A stent-in-stent approach using a 155 × 23-mm FCSEMS to improve coaptation also failed to resolve the fistula after 6 weeks.

Placement of a VAC Stent was proposed ( Video 1 ). During the procedure both FCSEMS were removed displaying purulent granulation tissue over the previous esophageal laceration ( Fig. 1 **a** ). A marking clip was placed 3 cm below at the gastric body and a metallic guidewire passed into the antrum. After saline irrigation, the introducer system was inserted transorally over-the-wire. The stent was successfully deployed under fluoroscopy ( Fig. 1 **b–d** ). The suction catheter was switched to the nose and connected to a vacuum pump at –120 mmHg during the first 24 h and then adjusted to –80 mmHg. Irrigation with 40 mL saline 3 times per day and starting on liquid diet after 72 h were advised. Stent removal was scheduled after 7 days with the pump being switched off the day before. The stent was detached by gently insinuating the endoscope between the stent and esophageal wall while irrigating profusely with saline. It was then removed by grasping the wire at the proximal end ( Fig. 2 ). Esophageal inspection revealed extensive granulation without extravasation of contrast ( Fig. 3 ). Control CT confirmed successful closure of the fistula ( Fig. 4 ) and the now asymptomatic patient was discharged.

**Fig. 1:**
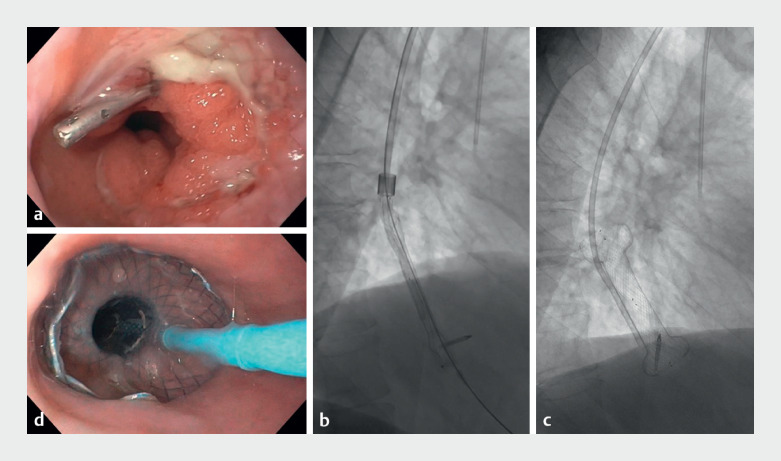
**a** Purulent granulation tissue over the previous esophageal laceration in a patient with Boerhaave syndrome. **b, c** Deployment of the innovative hybrid VAC Stent under fluoroscopic view. **d** Endoscopic view of the deployed stent.

**Fig. 2:**
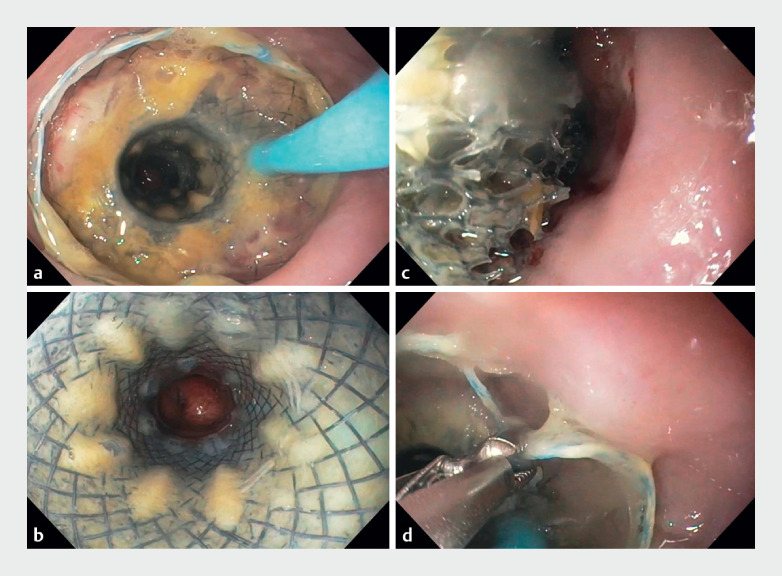
**a, b** The novel hybrid stent in situ. **c** The stent is detached by gently insinuating the endoscope between the stent and the esophageal wall while irrigating profusely with saline. **d** Removal of the stent by grasping the wire at the proximal end.

**Fig. 3:**
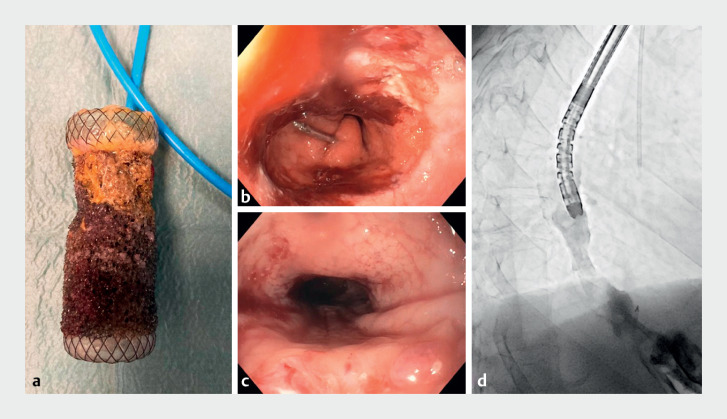
**a** The vacuum therapy stent after removal. **b, c** Endoscopic inspection after stent removal revealed extensive and friable granulation tissue. **d** Fluoroscopic control revealed no extravasation of contrast.

**Fig. 4:**
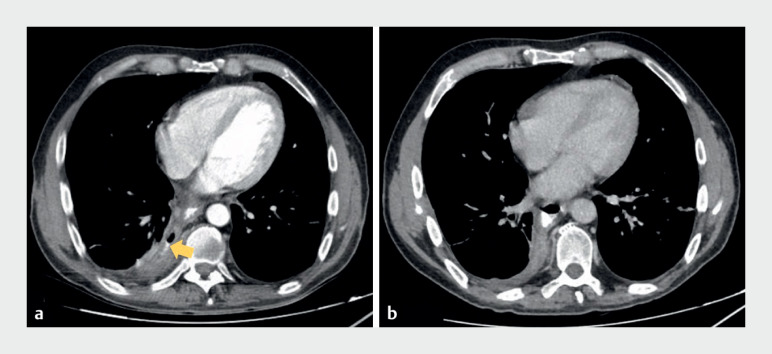
Computed tomography (CT) images. **a** Before therapy, the persistent esophagopleural fistula (arrow) can be seen. **b** Successful closure of the fistula is confirmed after therapy with the novel vacuum stent.

Successful treatment, using an innovative hybrid vacuum stent, of a refractory esophagopleural fistula in a patient with Boerhaave syndrome.Video 1

The VAC Stent combines the functions of a SEMS with EVT, avoiding migration while maintaining luminal patency 2 3 4 . This case demonstrates its effectiveness for treating esophageal perforation.

Endoscopy_UCTN_Code_TTT_1AO_2AZ

## Notes

Endoscopy E-Videos https://eref.thieme.de/e-videos
*E-Videos* is an open access online section of the journal *Endoscopy* , reporting on interesting cases and new techniques in gastroenterological endoscopy. All papers include a high-quality video and are published with a Creative Commons CC-BY license. Endoscopy E-Videos qualify for HINARI discounts and waivers and eligibility is automatically checked during the submission process. We grant 100% waivers to articles whose corresponding authors are based in Group A countries and 50% waivers to those who are based in Group B countries as classified by Research4Life (see: https://www.research4life.org/access/eligibility/ ). This section has its own submission website at https://mc.manuscriptcentral.com/e-videos .
